# Social Undermining and Interpersonal Rumination among Employees: The Mediating Role of Being the Subject of Envy and the Moderating Role of Social Support

**DOI:** 10.3390/ijerph19148419

**Published:** 2022-07-10

**Authors:** Ying Song, Zhenzhi Zhao

**Affiliations:** 1 School of Economics and Management, China University of Petroleum (East China), Qingdao 266580, China; sy-0129@163.com; 2 Business School, Qingdao University of Technology, Qingdao 266520, China; 3 Nanyang Business School, Nanyang Technological University, Singapore 639798, Singapore

**Keywords:** rumination, interpersonal rumination, supervisor social undermining, co-worker social undermining, subject of envy, workplace social support, psychological well-being

## Abstract

Rumination is a common problem and is associated with reduced psychological well-being. However, little is known about how rumination in the workplace is affected by interpersonal relationships. We propose that negative workplace behavior could serve as a potential influencing factor for rumination. Therefore, the current study constructed a multilevel moderated mediation model to investigate the relationship between workplace unit social undermining and interpersonal rumination. We also examined whether unit social support moderated that relationship and whether being the subject of envy mediated that relationship. Survey data were collected from 630 employees in China. The results indicate that: (1) a high level of unit social undermining by either a supervisor or co-workers has a significant positive influence on interpersonal rumination; (2) being the subject of envy exerts a mediating effect between unit supervisor social undermining and interpersonal rumination, as well as between unit co-worker social undermining and interpersonal rumination; and (3) unit social support moderates the associations between unit supervisor/co-worker social undermining and interpersonal rumination. These findings extend the research on rumination to the field of management and interpersonal relationships and emphasize the potential mechanisms of rumination, providing significant guidance for reducing staff rumination and improving psychological well-being.

## 1. Introduction

A good state of mental and emotional health constitutes psychological well-being. Psychologically healthy people have no mental illnesses and learn to control their stress so that it does not affect their ability to enjoy life and contribute to society [1]. Though most people experience times when they are not mentally or emotionally at their best, being in a state of psychological well-being means that they are able to effectively deal with such challenges [2]. Psychological well-being can also impact one’s physical health. Kiken and Shook found that improving psychological well-being could influence one’s ability to deal with positive and negative outcomes [3]. Given the importance of psychological well-being to one’s mental and physical health, it is essential to explore ways to prevent poor psychological well-being.

One factor that researchers have found to be associated with low psychological well-being is excessive rumination [4,5]. Rumination is defined as a mode of recurrent thoughts that occur in response to negative emotions or life experiences [6]. The tendency to ruminate has been empirically linked to emotional disturbance, which refers to the exaggeration, confusion, and decline of normal emotional responses [7]. Rumination can affect one’s ability to block out non-essential information while processing data, as well as one’s ability to integrate data from multiple sources [8]. It is also associated with anxiety, depression, shame, inattention, anger, pessimism, and a reduction in one’s sense of control over one’s life [9]. Furthermore, evidence suggests that rumination may be important in maintaining negative mood states after interpersonal confrontations [10]. Rumination is thus an important reflection of one’s mental well-being and psychological functioning.

Most studies on rumination have concentrated on the individual characteristics associated with the tendency to engage in rumination [11]. Prior research has shown that the antecedents of rumination include stressful life events such as peer victimization, neglect, and emotional abuse [12]. Parental influence has also been shown to affect rumination [13], particularly with regard to negative feedback from parents, parents’ tendency to practice overprotective and over-controlling parenting [14], and physical and emotional ill-treatment experienced during childhood [15].

It is important to note that ruminative behavior is not exclusive to individuals suffering from childhood abuse or other traumatic life events. Indeed, various daily events may trigger rumination in individuals. This is especially true when people are confronted with conditions that cause them to feel angry, agitated, or depressed. For example, control theory defines rumination as recurrent instrumental thoughts about unsatisfactory aim development and suggests that individuals who believe that they are making slower-than-expected progress toward a goal are more likely to engage in rumination until they either make progress towards its achievement or abandon the goal [16]. This suggests that rumination is not necessarily pathological, but rather, rumination is an adaptive response to daily difficulties [13]. Hence, rumination can be disruptive if it does not help individuals to either make progress towards or disengage from their goals and instead only intensifies negative feelings about their inability to achieve their goals [17].

The focus of the current literature on rumination as a fundamental individual trait does not help us to understand how rumination can arise in response to events in one’s daily life. It also represents a squandered chance to learn more about the function of ruminative thinking in helping people to overcome interpersonal struggles in their everyday lives. We wish to supplement current research by exploring the factors that may affect rumination, particularly in the workplace. Studies have found that rumination makes individuals more likely to endorse negative, prejudiced perceptions of situations, resulting in less effective solutions to interpersonal issues [18]. For example, previous research has shown that rumination interferes with successful problem-solving because it makes people more gloomy, as well as more abstract and less capable of accessing particular details about how to address a problem [19].

Negative rumination is associated with significant detrimental effects on various aspects of an individual’s life [20]. Individuals engaging in negative ruminative thinking have been found to experience greater work stress [21] and work-related fatigue [22], behavioral changes such as diminished sleep quality [23] and greater alcohol use [24], and negative outcomes such as a decrease in job performance [25] and worsening health [26]. It is, therefore, important to understand what affects rumination in the workplace.

Few studies have focused on understanding rumination in daily life. Hence, we build on the work of Wade et al. [27], who devised a genuine and reliable scale for measuring rumination in the aftermath of an interpersonal offense. In extending this construct and applying it to the workplace, we are able to see the potential of bringing rumination from the domain of psychology into the management literature to understand what contributes to employee rumination. This can help to clarify the relationship between rumination and employees’ ability to tackle and overcome unfavorable situations, as well as help us to understand how to create a more conducive environment to ensure employee well-being.

In the current study, we specifically examine interpersonal rumination, which is defined as the continuous, repetitive rehearsal of interpersonal problems arising from events and situations in which individuals are hurt by interpersonal encounters [27]. In the workplace, an individual may encounter interpersonal problems that challenge their perceptions of personal safety and control. Unfriendly, unexpected, or hurtful interpersonal interactions can overwhelm an individual and result in heightened rumination about a particular occurrence, regardless of one’s proclivity for ruminating [27]. For example, Lissnyder highlighted the fact that stress arising from job insecurity in the workplace is likely to increase work-related rumination [28]. According to preliminary research, workplace mistreatment and rumination are positively correlated [29]. Wang et al. discovered that when staff were subjected to more client abuse, they were more likely to ruminate on their unfavorable experiences at night [30]. Ruminating about uncivil workplace encounters can have a negative effect on a person’s career, for example, by lowering job satisfaction or increasing the likelihood of job burnout. Non-work outcomes can also be harmed, resulting in decreased life satisfaction and interference with non-work duties [31]. Hence, interpersonal rumination is not only a key indicator of employee well-being but also an important factor influencing one’s continued ability to engage efficiently and positively with one’s work.

In the current study, we explore how social factors affect interpersonal rumination in the workplace. The supervisor–co-worker connection has long been seen to be important in understanding and determining how people behave in work units or teams. Such a relationship, depending on its nature, is likely to have either a beneficial or a harmful impact on work behavior (i.e., supportive vs. undermining) [32]. An employee’s relationships with managers and co-workers in the same unit are the two dominant social relationships that define the work environment [33]. Since managers operate as representatives of the company, employees interpret their behavior toward them as an indication of how much the company values their contributions and cares about their well-being [34,35]. Managers are also a valuable source of advice, help, and feedback for subordinates as they complete their job duties [36]. As stated by Boh, co-workers’ behavior provides a main source of information, telling workers how a person should adapt and what kind of behavior will pay off in the larger unit [33]. An employee’s interactions with his/her manager and co-workers are immediate and frequent. In this research, we explore how perceptions about social relationships with one’s supervisor and co-workers affect interpersonal rumination.

The current study contributes to the existing literature in the following ways. First, past research has focused on rumination in clinical psychology by examining how rumination is associated with clinical depression, as can be seen in the literature on how parents, childhood experiences, and a family history of mental health difficulties affect rumination. As highlighted previously, rumination can also result from daily life events and interpersonal relationships and is an important aspect of employee well-being. Hence, we take the concept of interpersonal rumination from the clinical psychology domain into the management domain in order to understand what factors influence interpersonal rumination among employees in work settings. Second, we focus on social factors in work settings as antecedents of rumination. Given that employees’ social environments are largely influenced by unit supervisors and co-workers, we analyze how negative and positive elements of social interaction with these individuals affect rumination. This allows us to look into the theoretical underpinnings of previous studies that have emphasized the role of interpersonal rumination in units. Third, we identify the mediating mechanism of how negative social interactions in workplace units affect rumination. This focuses on how unit supervisors and co-workers can influence employees’ emotions and their feelings about how others view them, thus influencing interpersonal rumination. This is significant because it has the potential to explain the theoretical mechanism by which perceived negative behaviors from managers and co-workers influence rumination.

### 1.1. Interpersonal Rumination

Rumination is repeated, lengthy, and recurrent negative thinking about one’s thoughts, personal worries, and upsetting experiences [37]. Rumination appears to have a negative influence on sentiment and sentiment-related cognition. Existing emotional states such as sadness, anger [38], anxiety [39], and despair can be exacerbated and prolonged by rumination [40,41]. Ruminative thinking can also cause individuals to elaborate upon and further polarize the thought content that they focus upon during rumination [17]. Rumination has a magnifying effect because it enhances self-focus, exacerbating the vicious cycle of poor mood and negative cognition [42]. Rumination also draws attention to the disparity between one’s intended state and reality, emphasizing any mismatch.

Frone [43] defined unfavorable work rumination as a kind of worry and repetitive thinking about negative working conditions. People may ruminate over work-related worries and incidents when they are not at work. For example, in a previous survey of 3000 employees, 72% said they were concerned about their work while off-duty [20]. At work, however, interpersonal relationship problems are the most likely cause of rumination. When ruminating, people focus their mental attention on the negative experiences and unfavorable consequences of past events [27]. Interpersonal rumination, in other words, is a distressing and undesirable cognitive activity because it extends the focus on unfavorable interpersonal encounters. Interpersonal rumination is also a kind of stress reaction, which involves persistent and passive thinking on issues related to social interactions [44].

### 1.2. Social Undermining

Rumination is an evaluation of the coping process, and it can reflect interpersonal relationships. To better examine how behaviors reflect interpersonal relationships and influence rumination, we draw on the idea of negative behaviors. Negative behaviors in the workplace include inappropriate language, uncooperative work, improper communication tone, malicious competition, conflicts, and frame-ups. These behaviors overlap, but to different degrees. Therefore, in order to more comprehensively study negative behaviors at work, we need to investigate the influence of various negative behaviors at different degrees and frequencies. To do so, we draw on the concept of social undermining.

Social undermining is defined as activity that seeks to sabotage the development and maintenance of strong interpersonal connections, professional success, and a positive reputation [45]. Social undermining concerns a variety of common forms of negative behaviors through manners and verbal actions in the workplace. For example, direct actions may be used to undermine social norms, such as deliberately speaking ill of someone, completely rejecting someone, or playing down someone’s views. Undermining may also be realized by concealment, such as concealing necessary information or not defending someone in a conflict [46]. Verbal undermining may include disparaging comments, keeping silent, or failing to transmit critical information to an intended recipient [47]. Social undermining causes significant disruptions to the victim’s social relationships, as employees perceive that their supervisors and co-workers intentionally target the individual [48], making the accomplishment of their work particularly challenging [49]. It is critical to understand victims’ reactions to social undermining, as undermining can cause a sense of confusion and threat amongst employees and increase their work stress [50]. Increased perceptions of being the target of undermining behavior can create significant negative emotions, especially when social undermining becomes too prevalent, creating highly unfavorable working conditions for the targeted employee [50].

In the workplace, social undermining can come from two key sources: supervisors and co-workers [47]. Supervisors and co-workers represent two important workplace relationships, as these relationships are significantly associated with one’s work performance and reputation in the workplace. Hence, employees are likely to have strong reactions when they perceive social undermining originating from these individuals [47]. Further, social undermining from supervisors and co-workers reflects the prevalent normative behaviors in the work unit and can create a negative working environment. Hence, it is important to examine the climate of social undermining by supervisors and co-workers in a work unit.

Supervisor undermining refers to individuals’ perceptions that supervisors exhibit behaviors that appear to hinder the ability of a subordinate to perform well, establish positive work relationships, and establish a positive reputation at work [51]. When employees show low efficiency, make mistakes, become involved in interpersonal conflicts, or experience disharmony with the supervisor’s personality, supervisor undermining may occur. Examples of social undermining by supervisors include explicit behavior, such as supervisors belittling subordinates, and implicit behavior, such as withholding important information or giving employees the “silent treatment.”

Co-worker undermining comprises actions by co-workers that are meant to obstruct one’s job and prevent one from establishing a positive reputation. Co-worker social undermining can occur due to workplace rivalry, interpersonal conflicts, unpleasant episodes of cooperation, or other negative events in the workplace. Examples of social undermining by co-workers include the spreading of rumors about colleagues, deliberately delaying one’s work to hold other employees back, or giving them wrong or misleading information. Supervisors and co-workers can also be motivated to undermine those who could threaten their future status, regardless of whether they pose a current threat [52].

Negative occurrences, particularly those that, like undermining, breach behavioral norms, are the main triggers of ruminative thinking [48]. According to equity theory, when people are confronted with unfavorable conditions such as social undermining, they will engage in ruminative thinking [39]. Their assessment of their current state is not only based on the actual facts but will also involve repeatedly thinking about what things should or could be [48]. Jeffrey Roelofs’s research has found that rumination is significantly associated with depression, trait anxiety, undermining, perfectionism, and narcissism [53]. Previous conflict research indicates that work team social conflict has a damaging impact on team members’ reactions and emotions, as well as employee satisfaction and psychological well-being [54]. The atmosphere of team conflict is related to employees’ bad moods and ruminate thinking after work [55]. Thus, when employees experience social undermining in their work unit, their tendency to ruminate will be affected [56].

We thus posit the following hypotheses:

**Hypothesis** **1** **(H1).** *Unit supervisor undermining will positively affect employees’ interpersonal rumination*.

**Hypothesis** **2** **(H2).** *Unit co-worker undermining will positively affect employees’ interpersonal rumination*.

### 1.3. Mediating Role of Subject of Envy

On the one hand, social undermining can directly influence interpersonal rumination, but, on the other hand, social undermining can also indirectly affect interpersonal rumination by affecting one’s emotions. This is because ruminative thinking involves repeated thinking about one’s interpersonal problems based on self-evaluation and comparisons with others in the workplace. We propose that social undermining makes emotions related to being compared to others more salient, thus leading to greater interpersonal rumination.

Social undermining triggers important emotions that individuals experience when they evaluate how they are treated and regarded by others. In particular, individuals’ emotions are deeply affected by their perceptions of the extent to which they are the subject of envy by others; we thus propose such perceptions as an important mediator in the relationship between social undermining and interpersonal rumination [57].

Prior research on envy has mostly focused on the influence of envy on the emotional reactions and behavioral choices of people who are envious of others, but little attention has been paid to the emotional reactions and behavioral choices of those who are the subject of such envy [58]. Of the few research studies that examined this perspective, some have highlighted the pain and negative social experiences of those subjected to others’ envy, highlighting how such comparisons affect employees’ emotional responses, behavioral choices, and work performance.

In the workplace, work units are places where employees compete for recognition, resources, promotion, and rewards [59], and they may become the subject of envy as a result of reaching these goals. On one hand, being the subject of others’ envy can be pleasurable: it can evoke feelings of self-improvement, satisfaction, superiority, and accomplishment [60]. On the other hand, because of harsh comparisons made by other employees, being the subject of envy can be unpleasant and undesirable [61] or involve painful negative emotions originating from envious colleagues [49,62].

In order to have good working efficiency, employees are generally required to be vigorous and absorbed [63]. However, employees may find it challenging to restrain their passion when faced with unpleasant interpersonal situations (such as being envied by others) [64]. Being envied will reduce the quality of employees’ work and damage their task performance and the working atmosphere. Gradually, the damage will increase to a much more severe level, such as reducing the performance of enterprises. Considering the widespread and harmful influence of being the subject of envy, it is important to study the mediation role of being the subject of envy between its cause and effect in the workplace. This would help researchers and managers to better understand the causes of being the subject of envy and how it damages the work results of the envied party, therefore allowing them to put forward effective viewpoints to reduce its occurrence and harmful effects [65].

Several studies have found that envious individuals often express their envy through hostile behaviors toward the targets of their envy. Research has found that these hostile behaviors can manifest as trying to destroy the future achievements of the target or attacking, slandering, and alienating them, sometimes leading to a hostile environment [66]. Similarly, Parrott found that the hostility, alienation, and coldness of envious individuals or environments, as well as the sarcastic and critical remarks that they make, often heighten the targets’ perceptions that they are the subject of others’ envy [67]. In one study, Silver showed a video of communication between a perceived superior party and a perceived inferior party to his subjects. He found that when the inferior party belittled and slandered the success of the superior party, the subjects reported that the inferior party was envious of the superior party. This shows that individuals judge whether one is the subject of others’ envy through the language and expressions of the envious party [68]. In another study, Mosquera asked subjects to recall a scene in which they had been the subject of envy by others and answer this question: “What do others do and say to show they envy you?” Through coding and content analysis, the researchers found that individuals can judge whether they are the subject of envy from the clues exhibited in others’ behavior, verbal language, and body language. These include unfriendly behaviors such as changes in how individuals communicate (i.e., a sharp tongue, unnatural gestures, and facial expressions) [60]. The unfriendly behaviors, hostile behaviors, and hostile atmosphere mentioned above are expressions of social undermining from either individuals or environments. Therefore, we propose that work unit social undermining has a positive predictive effect on one’s perception of being the subject of envy.

Researchers suggest that being envied may create a sense of alienation from others. If the envied person is concerned with interpersonal relationships, he or she can feel uneasiness, stress, tension, and frustration about interpersonal problems, all of which will gradually intensify. The work requirement-resource model suggests that employees’ perceptions of being envied are a kind of interpersonal pressure caused by their fear and anxiety about other people’s destructive behaviors, causing them to spend more psychological resources on uncontrollable rumination [69]. Therefore, individuals who are the subject of others’ envy will tend to engage more in interpersonal rumination.

Individuals are more likely to perceive clues that they are the subject of envy when they experience social undermining. Prior studies have described the range of stress, anxiety, self-blame, guilt, and distress experienced by individuals as a result of being the target of envy [63]. This suggests that those who are the target of envy will have negative experiences, such as uneasiness, worry, and chagrin, as they feel threatened by others [70]. Such negative emotions and experiences are heightened when the person who is the subject of others’ envy values interpersonal relationships [65,71]. In such situations, the subject of envy is more likely to keep dwelling on interpersonal problems arising from the envious individuals. The negative emotions generated will likely increase the stress that they face, as they repeatedly think about their interpersonal relationship problems with the envious party and repeatedly think about the cause and effect that led to such a relationship. This repetitive thinking constitutes interpersonal rumination. We thus propose:

**Hypothesis** **3a** **(H3a).** *Employees’ perceptions of being the subject of envy mediate the relationship between unit supervisors’ social undermining and employees’ interpersonal rumination*.

**Hypothesis** **3b** **(H3b).** *Employees’ perceptions of being the subject of envy mediate the relationship between unit co-workers’ social undermining and employees’ interpersonal rumination*.

### 1.4. Moderating Role of Unit Social Support

The effect of unit social undermining on interpersonal rumination is not stable. It will instead be influenced by other related factors. Social support and social undermining are two sides of the same coin: each reflects the positive and negative behaviors of co-workers. To better examine the effect mechanisms, we draw on the concept of social support. We aim to determine whether social support from one area (i.e., supervisors) can mitigate the negative effects from another area (i.e., co-workers.)

The stress buffer hypothesis provides a key theoretical basis for explaining the interaction between social support and undermining [72]. According to this hypothesis, the negative effects of stressors will be decreased for people who stay in a high social support environment [48]. To some extent, being undermined by one’s supervisor is stressful, but getting support from other people (colleagues or supervisors) in the same unit may cushion the harmful effects of such stress [73]. For example, De Fluiter found that supervisor support weakened the negative influence of supervisor abuse on employee satisfaction [74]. In addition, co-worker support has been found to be positively related to communicative responsiveness [75] and job performance [76]. In the workplace, people who have been repressed will exhibit a greater psychological reaction when they receive social support [47]. If social support and social undermining are both produced by the same unit, the social support will mitigate the negative impacts of the social undermining.

According to the theory of organizational support, employees pay attention to treatment provided by the organization to identify the degree of organizational support and the value of their contributions [77]. Researchers have proved that the social support of the organization promotes employees’ trust in the organization, thereby reducing their worries about work and interpersonal relationships [78,79]. Research that focused on Taiwan’s semiconductor industry found that team social support can influence the relationship between team stress and employee performance. Team stress derives from work duty and interpersonal relationships [80]. Research about high-level sports also found that team social support moderates the influence of team and culture stress on subjective behavior [81].

Equity theory suggests that in a unit or group, when the level of social support is low, individuals who are faced with negative situations (i.e., those experiencing social undermining from their co-workers or supervisors) will tend to experience counterfactual thinking in the form of rumination [48]. Rumination involves constantly strengthening and analyzing negative information, and thus, when the degree of social support is high, the source of rumination is small.

We thus posit the following hypotheses:

**Hypothesis** **4a** **(H4a).** *Unit social support moderates the relationship between unit supervisor social undermining and employees’ interpersonal rumination*.

**Hypothesis** **4b** **(H4b).** *Unit social support moderates the relationship between unit co-worker social undermining and employees’ interpersonal rumination*.

**Figure 1:**
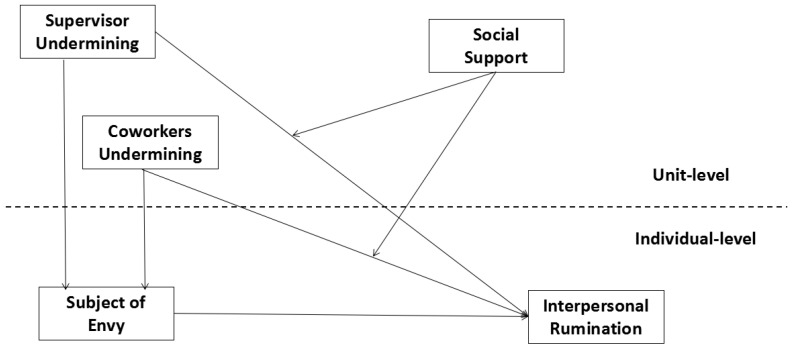
Hypothetical model.

Based on the above analysis, this study proposes a theoretical model of the multilevel effect of unit social undermining on an employee’s interpersonal rumination. This study also uses being the subject of envy as a mediator and unit social support as a moderating variable, as shown in Figure 1.

## 2. Materials and Methods

### 2.1. Model Development

Researchers usually estimate coefficients and influences through a single-level statistical model, such as ordinary linear regression or analysis of variance [82]. However, the standard deviation bias frequently occurs when processing multilevel data. As shown in Figure 1, we built a two-level variable model. The model for analyzing multilevel data is the Hierarchical Linear Model (HLM) [83]. By establishing multilevel regression equations, the error is decomposed into all levels of errors, which solves the problem of the independence of random errors. Thus, we can explore the influence of different levels of independent variables on dependent variables and the interaction between different levels of independent variables. Hence, we used HLMs in this study.

### 2.2. Participants

The current study began with a pilot survey to determine whether the scale was user-friendly and appropriate for investigating workplace conditions. The sample selected for the pilot survey was mainly based on the reliability of the sample and the convenience of the survey. Moreover, considering the diversity of samples, this study focused on executive staff and enterprise employees, so the choice of the pilot survey mainly focused on these two groups. To do this, we randomly sampled 207 university executive staff and enterprise employees in China to take part in the study online (on the Sojump website). The study sample included 72 university executive staff, 79 large enterprise employees, and 56 startup company employees. The results of the pilot survey show that there was no confusion for these items, and all of the respondents felt that the scale was appropriate for assessing workplace conditions. Moreover, comparison results showed that a reward of 5 US dollars could ensure the quality of the responses to a certain extent (i.e., the response was not a wide range of repeated answers and showed no obvious regularity). Thus, in order to improve the quality of the data, we gave each respondent 5 US dollars to participate in the survey.

We recruited employees from universities, government agencies, and enterprises in China to take part in the study online (on the Sojump website and email). For those who indicated their willingness, we invited them to invite their teammates to participate. Kreft found that when the number of groups is greater than 30, reliable parameter estimation can be obtained in a multilevel model [84]. As for the number of individual samples in a group, researchers found that when the number of groups is greater than 30 but less than 70, and the number of individual samples in the group is less than 20 but more than 5, the reliability of parameter estimation is better [85]. Thus, in this research, we randomly recruited 65 work units from universities, government departments, and enterprises to participate in the research. Of the 65 teams, 15 rejected the opportunity to take part. Participants who provided insufficient demographic information or incomplete responses to survey items were marked as invalid. We also excluded teams with very low response rates. Ultimately, the valid data comprised completed questionnaires from 630 employees across 53 teams. The study sample included 109 university executive staff, 95 civil servants, 169 large enterprise employees, 176 medium-sized company employees, and 81 startup company employees. The sample size of each unit ranged from 7 to 18. The study sample included 292 males and 338 females, with an average age of 33.6 years (ranging from 21 to 60 years) and an average annual salary equivalent to 14,260 US dollars. In the sample, 11% of participants reported a career length of less than one year, 28.6% reported 1–3 years, 16.3% reported 4–5 years, 24.2% reported 6–10 years, and 19.4% reported a career length of more than 10 years.

Participation was completely anonymous. During the research process, special attention was paid to avoiding potential physical or mental harm to the participants. In general, research participants have the potential to suffer psychological harm in the process of social research. Therefore, the researchers in this study were cautious and alert to even the smallest risk.

### 2.3. Measures

The scales used in this study were previously used in studies of English speakers. We used the traditional translation and back-translation technique to ensure the scales’ equivalency and content validity. All survey items were graded on a 7-point Likert scale ranging from 1 = strong disagreement to 7 = strong agreement.

#### 2.3.1. Interpersonal Rumination

Interpersonal rumination was measured using the 5-item scale developed by Wade [27]. This 5-item measure is designed to capture rumination as the repetitive rehearsal of specific past interpersonal offenses and continuous attention to the processes and results of negative events. Participants were prompted to recall interpersonal problems or their experiences of negative events in the workplace and then answer questions such as: “I cannot stop thinking about how my colleagues have misunderstood me,” “I can’t seem to get the images of how I was abused out of my head,” and “I attempt to find out why my co-workers are hurting me.” Higher scores indicate a higher level of rumination. The Cronbach’s alpha for this scale was found to be good (*α* = 0.854).

#### 2.3.2. Subject of Envy

The extent to which the respondent was the subject of envy was assessed using three items developed and validated by Vecchio [86]. Sample items included, “My co-workers occasionally resent me because of my professional achievement,” “My co-workers occasionally resent me because of the tight working relationship I have with my supervisor,” and “My achievements have made some of my co-workers envious.” The Cronbach’s alpha for this scale was found to be good (*α* = 0.888).

#### 2.3.3. Social Undermining

The 26-item version of the social undermining scale (SUS-26) was used to assess social undermining [47]. It takes into account both supervisor and co-worker social undermining (13 items each). Sample items for supervisor undermining (*α* = 0.931) were, “My supervisor frequently shows impatience when I question work procedures,” and “My supervisor frequently ignores my achievements and slows me down.” Sample items for co-worker social undermining (*α* = 0.940) were, “My co-workers frequently say something to upset me,” and “My co-workers frequently give me the silent treatment.” At the unit level, both supervisor and co-worker undermining were quantified as components of a negative work climate. As a result, the unit-level variable was calculated as the average level of supervisor and co-worker social undermining across all unit employees. The higher the score, the more social undermining there was in the unit.

#### 2.3.4. Social Support

We adopted the social support scale following Zimet’s Multidimensional Scale of Perceived Social Support [87]. Sample items were, “My supervisor frequently stands in my shoes when I face trouble with my work,” and “My co-worker frequently cares about my emotional status.” Internal consistency was found to be high in the sample (*α* = 0.868). Social support was also measured at the unit level as a marker of positive unit work climate. The unit-level variable was calculated by averaging all of the team members’ perceptions of social support. A higher value indicated that the unit had a higher level of social support.

## 3. Results

### 3.1. Common Method Bias Test

Harman’s single factor test was used to test for common method bias. We discovered that the largest variance factor’s explanatory fraction was 27.6%, which revealed no common method bias.

We also used the multitrait–multimethod (MTMM) matrix to test for common method bias. Table 1 shows the results for the common method bias test through the structural equation model (Δ*χ*^2^ = 67.428, Δ*df* = 55, *p* > 0.05), indicating that there was no common method bias.

### 3.2. Correlation Analysis

The mean value, standard deviations, and correlations of all variables studied are shown in Table 2. There were significantly positive relationships between unit supervisor social undermining and subject of envy, as well as unit co-worker social undermining and interpersonal rumination. Moreover, there were significantly negative relationships between unit social support and other variable mentioned above.

### 3.3. Reliability and Validity Testing

We investigated intraclass correlation and the within-group agreement index (r_wg_) to determine whether it was necessary to aggregate the results at the individual level to generate unit-level scores (intraclass correlation coefficient, ICC). The r_wg_ value indicates how closely group members’ survey replies resembled each other, beyond what would be predicted by chance [88]. The ICC represents the unit’s dependability. Table 3 shows the ICC and r_wg_ for all data. The aggregation of the measures at the unit level was justified because all of the values were within acceptable limits.

To ensure accurate and reliable results, we first evaluated the scales’ validity and reliability. An exploratory factor analysis (EFA) revealed that all of the KMO values were greater than 0.7, and the item communalities were greater than 0.6, indicating that the data were eligible for EFA (Zhu et al., 2019)

Cronbach’s alpha and composite reliability (CR) ratings were used to examine the scale’s reliability. All values for the two indicators were derived via an analysis using SPSS 22.0 and Mplus 7.4 software. Table 4 shows that each factor’s Cronbach alpha and CR value were greater than 0.7, indicating that the scale utilized in this investigation was reliable [89].

The average variance extracted (AVE) was utilized to assess our scale’s convergent validity (see Table 4). All of the variables had AVE values of more than 0.5, which we consider significant evidence of convergent validity. Furthermore, confirmatory factor analysis (CFA) was utilized to assess the scale’s discriminant validity, from which a well-fitting model was derived. The *χ*^2^/*df* in the CFA value for each scale was smaller than or nearly 5, while the CFI and TLI were greater than 0.9, indicating that the validity was good (see Table 4).

### 3.4. Analyses of the Main Effect and Mediating Effect

Using Mplus 7.4 software, we employed structural equation modeling to assess our hypotheses. The fit indices’ values were all within the allowed ranges (*χ*^2^/*df* = 4.203, *CFI* = 0.985, *TLI* = 0.961, and *RMSEA* = 0.073), indicating that the model’s final fit was satisfactory.

**Figure 2:**
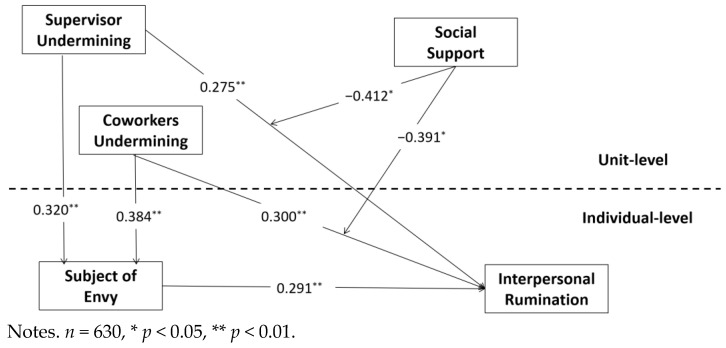
Hypothesis testing results.

The standardized path coefficients of the model are shown in Table 5 and Figure 2. After controlling the statistical characteristics, unit supervisor social undermining (Model 4, *β* = 0.275, *p* < 0.01), unit co-worker social undermining (Model 4, *β* = 0.300, *p* < 0.01), and experience of being the subject of envy (Model 4, *β* = 0.291, *p* < 0.01) were all found to have a significant positive impact on interpersonal rumination. Unit supervisor social undermining (Model 4, *β* = 0.320, *p* < 0.01) and unit co-worker social undermining (Model 4, *β* = 0.384, *p* < 0.01) both had a significant positive impact on experiences of being the subject of envy. H1 is thus supported.

### 3.5. Mediation Effect Test

We used Mplus 7.4 software to analyze the two mediation effects using the bootstrapping method. The sample size for the repeated bootstrap was set to 2000. We found that the mediating effects of being the subject of envy were all significant (see Table 6). Thus, H3 is supported.

We also tested the mediation effect of envy, but the fitting result was not good. Thus, only being the subject of envy can mediate the relationship between unit social undermining and individual interpersonal rumination.

### 3.6. Moderating Effect Test

In support of H3, we found that the coefficient of the interaction between unit supervisor social undermining and unit social support was negative and significant (*b* = −0.412, *p* = 0.004), as shown in Figure 2. Similarly, the interaction between unit co-worker social undermining and unit social support had a significant negative correlation coefficient (*b* = −0.391, *p* = 0.016).

In Table 7, the interaction between unit social support and unit supervisor social undermining can be seen to have a negative coefficient (*b* = −0.412, *p* = 0.004, *CI*(−0.694, −0.129)), indicating a negative moderating effect of unit social support on the relationship between unit supervisor social undermining and interpersonal rumination. The coefficient of the interaction between unit social support and unit co-worker social undermining is significantly negative (*b* = −0.391, *p* = 0.016, *CI*(−0.707, −0.075)), demonstrating a negative moderating effect of unit social support on the relationship between unit co-worker social undermining and interpersonal rumination.

**Figure 3:**
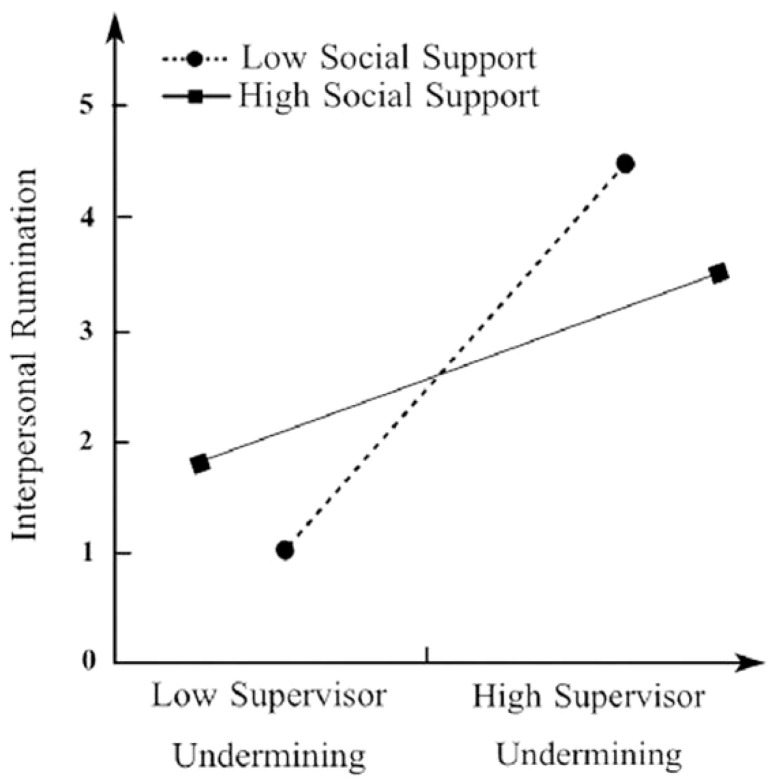
Interactive effect of unit supervisor social undermining and unit social support on interpersonal rumination.

**Figure 4:**
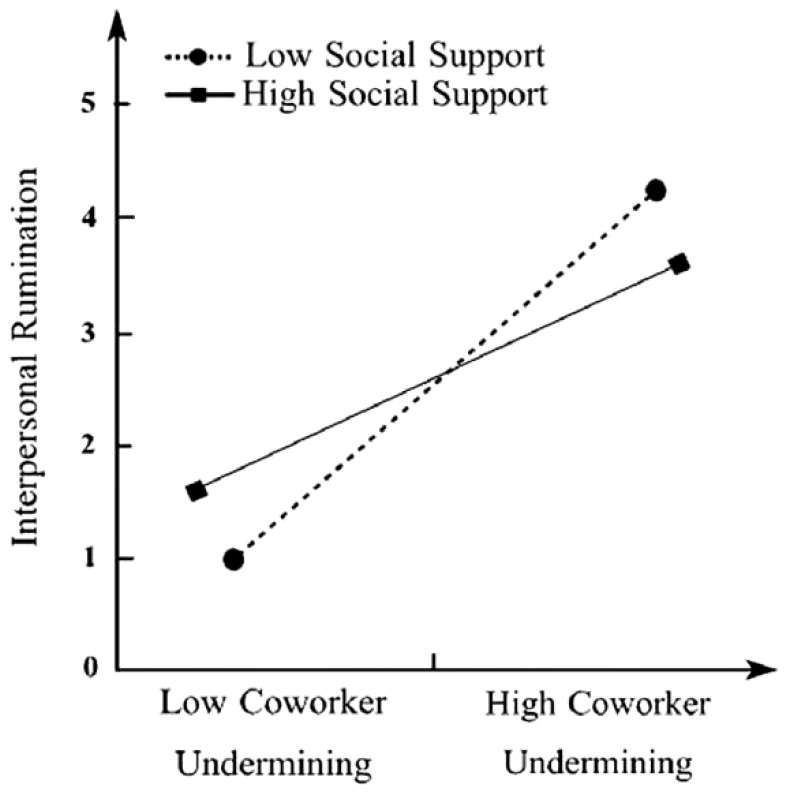
Interactive effect of unit co-worker social undermining and unit social support on interpersonal rumination.

Figure 3 and Figure 4 show that when unit social support was low, as unit supervisor/co-worker social undermining increased, the increase in interpersonal rumination behavior was greater than under the condition of high unit social support. This indicates that the effect of unit supervisor/co-worker social undermining on interpersonal rumination becomes stronger when the level of unit social support is lower.

This result shows that unit social support plays a moderating role between unit supervisor/co-worker social undermining and individual-level interpersonal rumination. Therefore, we selected the two forms of social support (supervisor social support and co-worker social support) to mirror unit social support and then conducted a robust test on the entire model. The result shows that unit supervisor social support moderates the relationship between both unit supervisor and co-worker social undermining and employees’ interpersonal rumination. Unit co-worker social support only moderates the relationship between unit supervisor social undermining and individual-level interpersonal rumination. These results verify that unit social support is a moderator in the entire model. Thus, H4 is supported.

## 4. Discussion

To examine negative behavioral influences on interpersonal rumination in the workplace, we developed a theoretical model to investigate the impact of unit social undermining, the mediating effect of experiences of being the subject of envy, and the moderating effect of unit social support. This research yielded three significant and innovative outcomes. First, unit social undermining was found to exert a significant positive influence on individual interpersonal rumination. Specifically, employees’ interpersonal rumination was significantly influenced by unit supervisor and unit co-worker social undermining. We contend that a more negative behavioral climate encourages individuals to focus on negative interpersonal conflicts, resulting in rumination. Individuals who see the corporate climate as hostile to interpersonal relationships are more likely to engage in repetitive and recursive thoughts in response to unfavorable behavior, resulting in interpersonal rumination. This indicates that unit social undermining has a particularly large impact on individuals’ interpersonal rumination.

Second, experiences of being the subject of envy were found to play a mediating role between unit social undermining and individual interpersonal rumination. In the workplace, when employees perceive a more unfriendly climate created by supervisor and co-worker social undermining, they will experience more feelings of being the subject of envy, resulting in more interpersonal rumination.

Third, unit social support played a moderating role between unit social undermining and individual interpersonal rumination. Specifically, when unit social support was low, the influence of unit social undermining on individual interpersonal rumination was more significant than when unit social support was high. Hence, individual interpersonal rumination is influenced more by unit social undermining when unit social support is low. In other words, for the employee, unit social support may compensate for the harm of social undermining, thus helping to reduce the negative influence of unit social undermining on individual interpersonal rumination.

### 4.1. Theoretical Implications

This study makes three major contributions to the literature. First, our research broadens the study of rumination into the management domain to explain what factors influence rumination among employees in the workplace. Prior studies have acknowledged the importance of negative behaviors and emotions to rumination [90] but have ignored the sources of clues that people depend on in the workplace. These early studies also tended to focus on psychopathology or individual characteristics [91,92] instead of explicitly assessing whether daily working life influences individuals’ rumination. However, rumination can also result from daily life events and is an important aspect of employee psychological well-being. Thus, it is necessary to study rumination in the domain of daily working life.

Second, we focused on social factors in work settings as antecedents of rumination. Employees’ social environments are largely influenced by their unit supervisors and unit co-workers. We examined how negative and positive elements of social interactions with these individuals affect rumination. Research on the positive elements has gradually matured [75,93], but research on the negative element of social undermining is relatively rare. This study introduced the variable of social undermining to the study of workplace interpersonal rumination. In addition, we used multilevel analysis to emphasize the importance of the unit and found that unit managers and co-workers serve as important social references in influencing individuals’ personal rumination, further influencing employees’ psychological well-being.

Third, based on the analysis results, social undermining can indirectly influence interpersonal rumination through the subject of envy. This explains the theoretical mechanism by which perceived negative behaviors from managers and co-workers influence rumination. This finding suggests that one’s social environment affects how individuals feel based on how they are perceived by others, which in turn affects rumination. In addition, previous research has only focused on envy as an emotion [94], while our study examined the perspective of an individual who is the subject of envy, enriching the literature on envy.

### 4.2. Practical Implications

This study has practical and organizational implications for how to effectively decrease interpersonal rumination among employees. Our study provides guidance to help managers reduce employees’ interpersonal rumination, create a harmonious working environment, and improve workplace productivity. First, in a workplace environment, managers must improve the treatment of employees to optimize the effects of a positive atmosphere on employees’ experiences. In particular, managers must reduce the output of negative behaviors, create a comfortable working environment, reduce interpersonal conflicts, and help employees to reduce stress and adjust their mentality. In addition, leaders should be aware of role modeling. At work, if leaders can be friendly, they can solve problems positively, increase supportive behaviors, reduce restraining behaviors, and reduce the spread of negative emotions. This will make employees more willing to work harder for team harmony and workplace productivity [95]. Finally, leaders should treat every employee fairly, judge employees fairly according to their performance at work, and help employees without reservation.

More importantly, our findings suggest that co-worker groups have an impact on people’s interpersonal rumination. These results suggest that employees should be encouraged to be friendly to others in the workplace. If they engage in positive behaviors to enhance peer consciousness of their actions and the rewards that come with them, then the likelihood that others will mimic their actions will rise.

Lastly, employees should keep a low profile when they are better than others or reach a point that others cannot. As interpersonal conflicts at work largely depend on one’s attitudes and behavior [96], when one’s words and actions reveal too much pride in being a winner, it will have an adverse effect on interpersonal relationships.

## 5. Conclusions

In the current study, we constructed a multilevel model to examine whether interpersonal rumination can be influenced by work unit social undermining. We theoretically and empirically demonstrated that workplace supervisor and co-worker social undermining have a significant positive influence on employees’ interpersonal rumination. Furthermore, we found that the influence of unit social undermining on interpersonal rumination is complicated and subtle. Employees’ subjects of envy have a mediating effect between unit social undermining and employees’ interpersonal rumination. Further unit social support plays a moderate mediating role in this relationship, too. This study extends rumination research into daily work life, interpersonal relationships, and the organizational climate. It also introduces the concept of social undermining in relation to workplace rumination and enriches emotion research regarding envy by exploring the importance of being the subject of envy. Our research provides guidance for managers to reduce employees’ interpersonal rumination, improve psychological well-being, and create a harmonious working environment.

## 6. Limitations and Future Research

This study has three limitations that should be addressed. First, the selection of units was divided into teams of employees working together, without considering the organizational culture of the company. If there is cooperation or competition between teams, other teams have the potential to also have an impact on individual employees. Therefore, we could add a third layer (e.g., company variables or relevant control variables) to the multilevel analysis to make the results more accurate.

Second, because we used data from a cross-sectional survey, causality could not be determined. An ideal study would track and focus on the behavior of individuals and their key reference persons to effectively evaluate the influence of key reference persons’ behaviors over a period of time. Therefore, longitudinal studies should be used in the future to give additional stronger support for these results.

Finally, our research sample was focused on Chinese employees, and the diversity of samples was insufficient. This limits the generalizability of the study findings to other populations. In terms of future research regarding the global impact of job burnout, it will be beneficial to conduct research on a more extensive and diverse group in order to ensure greater applicability, investigate better solutions to improve employees’ mental health and well-being, and further improve individual employee productivity.

## Figures and Tables

**Table 1:** Common method bias test.

Model	*χ* ^2^	*df*	*p*
1	2994.230	1259	
2	3061.658	1314	
Δ	67.428	55	>0.05

**Table 2:** Means, standard deviations, and intercorrelations of continuous variables.

Variable	*M*	*SD*	1	2	3	4	5
Unit supervisor social undermining	1.538	0.258	1				
Unit co-worker social undermining	1.442	0.227	0.868 **	1			
Unit social support	5.295	0.223	−0.354 **	−0.403 **	1		
Subject of envy	2.930	1.321	0.329 **	0.388 **	−0.127 *	1	
Interpersonal rumination	2.683	0.714	0.308 **	0.335 **	−0.289 **	0.317 **	1

Notes. *n* = 630, * *p* < 0.05, ** *p* < 0.01.

**Table 3:** ICC and rwg results of some indicators.

	ICC	r_wg_
Supervisor social undermining	0.792	0.9415
Co-worker social undermining	0.757	0.9328
Subject of envy		0.8146
Social support		0.7492

Notes. ICC = intraclass correlation coefficient.

**Table 4:** Statistical results of some indicators.

Variable	Cronbach’s Alpha	KMO	AVE	CR	*χ*^2^/*df*	*CFI*	*TLI*	N of Items
Supervisor social undermining	0.931	0.925	0.5301	0.9356	2.28	0.931	0.914	13
Co-worker social undermining	0.94	0.934	0.5502	0.9407	1.917	0.942	0.931	13
Subject of envy	0.888	0.745	0.7271	0.8887	0	1	1	3
Social support	0.868	0.747	0.5796	0.8458	3.201	0.943	0.921	4
Interpersonal rumination	0.854	0.885	0.5724	0.8423	3.493	0.972	0.94	6

Notes. KMO = Kaiser–Meyer–Olkin; AVE = average variance extracted; CR = composite reliability.

**Table 5:** Hierarchical regression analysis results.

Variable	Subject of Envy		Interpersonal Rumination	
	Model 1	Model 2	Model 3	Model 4
Gender	0.11	0.09	0.068	0.036
Age	0.065	0.033	0.081	0.062
Career length	−0.049	0.036	−0.176 *	−0.162
Salary	−0.124	−0.116	−0.123	−0.086
Unit supervisor social undermining		0.320 **		0.275 **
Unit co-worker social undermining		0.384 **		0.300 **
Subject of envy				0.291 **
*R* ^2^	0.032	0.129	0.051	0.133
Δ*R*^2^	—	0.097	—	0.82
*F*	2.045	7.274 **	3.336 *	7.58 **

Notes. *n* = 630, * *p* < 0.05, ** *p* < 0.01.

**Table 6:** Indirect effects.

Indirect Effect	Estimate	*p*
Unit supervisor undermining→ subject of envy→ interpersonal rumination	0.417	<0.01
Unit co-worker undermining→ subject of envy→ interpersonal rumination	0.346	<0.01

**Table 7:** The moderating effects of unit social support.

Variable	Interpersonal Rumination	
	Model 3	Model 5
Gender	0.068	0.107
Age	0.081	0.006
Worktime	−0.176	−0.055
Salary	−0.123	−0.043
Unit supervisor social undermining		2.707 **
Unit co-worker social undermining		2.757 **
Unit supervisor social undermining * Social support		−0.412 *
Unit co-worker social undermining * Social support		−0.391 *
*R* ^2^	0.051	0.160
Δ*R*^2^	—	0.109
*F*	3.336 *	5.807 **

Notes. *n* = 630, * *p* < 0.05, ** *p* < 0.01.

## Data Availability

The data presented in this study are available on request from the corresponding author.

## References

[1] Ding H., Yu E. (2021). Strengths-Based Leadership and Employee Psychological Well-Being: A Moderated Mediation Model. J. Career Dev..

[2] Perrins S.P., Varanasi U., Seto E., Bratman G.N. (2021). Nature at work: The effects of day-to-day nature contact on workers’ stress and psychological well-being. Urban For. Urban Green..

[3] Grégoire S., Doucerain M., Morin L., Finkelstein-Fox L. (2021). The relationship between value-based actions, psychological distress and well-being: A multilevel diary study. J. Contextual Behav. Sci..

[4] Jennifer W. (2015). Cultivating multiple aspects of attention through mindfulness meditation accounts for psychological well-being through decreased rumination. J. Psychol. Res. Behav. Manag..

[5] Fireman G.D. (2021). Psychological Wellbeing, Worry, and Resilience-Based Coping during COVID-19 in Relation to Sleep Quality. Int. J. Environ. Res. Public Health.

[6] Jing L.A., Wei W.B., Qian H.C., Pw D., Li L.D., Suo J. (2021). The relationship between phubbing and the depression of primary and secondary school teachers: A moderated mediation model of rumination and job burnout. J. Affect. Disord..

[7] Colombo D., Serino S., Suso-Ribera C., Fernández-Lvarez J., Botella C. (2021). The Moderating Role of Emotion Regulation in the Recall of Negative Autobiographical Memories. Int. J. Environ. Res. Public Health.

[8] Joubert A.E., Grierson A.B., Chen A.Z., Moulds M.L., Newby J.M. (2021). Managing rumination and worry: A pilot study of an internet intervention targeting repetitive negative thinking in Australian adults. J. Affect. Disord..

[9] Zhou H.X., Chen X., Shen Y.Q., Li L., Yan C.G. (2019). Rumination and the default mode network: Meta-analysis of brain imaging studies and implications for depression. NeuroImage.

[10] McCullough M.E., Orsulak P., Brandon A., Akers L. (2007). Rumination, fear, and cortisol: An in vivo study of interpersonal transgressions. Health Psychol..

[11] Fang L., Marchetti I., Hoorelbeke K., Koster E.H.W. (2019). Do daily dynamics in rumination and affect predict depressive symptoms and trait rumination? An experience sampling study. J. Behav. Ther. Exp. Psychiatry.

[12] Gu H., Ma P., Xia T. (2020). Childhood emotional abuse and adolescent nonsuicidal self-injury: The mediating role of identity confusion and moderating role of rumination. Child Abus. Negl..

[13] Watkins E.R., Roberts H. (2020). Reflecting on rumination: Consequences, causes, mechanisms and treatment of rumination. J. Behav. Res. Ther..

[14] Ying J.F., You J.N., Liu S.H., Wu R.Y. (2021). The relations between childhood experience of negative parenting practices and nonsuicidal self-injury in Chinese adolescents: The mediating roles of maladaptive perfectionism and rumination. Child Abus. Negl..

[15] Zhu W., Chen Y., Xia L.X. (2020). Childhood maltreatment and aggression: The mediating roles of hostile attribution bias and anger rumination. Personal. Individ. Differ..

[16] Roberts H., Watkins E.R., Wills A.J. (2013). Cueing an unresolved personal goal causes persistent ruminative self-focus: An experimental evaluation of control theories of rumination. J. Behav. Ther. Exp. Psychiatry.

[17] Watkins E.R., Nolen-Hoeksema S. (2014). A habit-goal framework of depressive rumination. J. Abnorm. Psychol..

[18] Lyubomirsky S., Nolen-Hoeksema S. (1995). Effects of self-focused rumination on negative thinking and interpersonal problem solving. J. Personal. Soc. Psychol..

[19] Dickson J.M., Moberly N.J., Huntley C.D. (2019). Rumination selectively mediates the association between actual-ideal (but not actual-ought) self-discrepancy and anxious and depressive symptoms. Personal. Individ. Differ..

[20] He Y., Walker J.M., Payne S.C., Miner K.N. (2021). Explaining the negative impact of workplace incivility on work and non-work outcomes: The roles of negative rumination and organizational support. Stress Health.

[21] Koster E.H.W., De Lissnyder E., De Raedt R. (2013). Rumination is characterized by valence-specific impairments in switching of attention. Acta Psychol..

[22] Mark C., David P., Davide M., Stefan S., Ilke I., Geoff T., Chris C. (2017). The Association between Work-Related Rumination and Heart Rate Variability: A Field Study. Front. Hum. Neurosci..

[23] Gómez-Odriozola J., Calvete E. (2021). The role of dispositional mindfulness profiles as predictors of sleep problems through rumination in adolescents over time. Personal. Individ. Differ..

[24] Wolitzky-Taylor K., Sewart A., Zinbarg R., Mineka S., Craske M.G. (2021). Rumination and worry as putative mediators explaining the association between emotional disorders and alcohol use disorder in a longitudinal study. Addict. Behav..

[25] Türktorun Y., Weiher G.M., Horz H. (2020). Psychological detachment and work-related rumination in teachers: A systematic review. Educ. Res. Rev..

[26] Mazzer K. (2018). A longitudinal view of rumination, poor sleep and psychological distress in adolescents. J. Affect. Disord..

[27] Wade N.G., Vogel D.L., Liao K.Y.-H., Goldman D.B. (2008). Measuring state-specific rumination: Development of the Rumination about an Interpersonal Offense Scale. J. Couns. Psychol..

[28] Liu M.X., Wang N., Wang P.C., Wu H.M., Ding X.E., Zhao F.Q. (2021). Negative Emotions and Job Burnout in News Media Workers: A Moderated Mediation Model of Rumination and Empathy. J. Affect. Disord..

[29] Schilpzand P., De Pater I.E., Erez A. (2016). Workplace incivility: A review of the literature and agenda for future research. J. Organ. Behav..

[30] Wang M., Liao H., Kammeyer-Mueller J., Liu S., Gong Y., Shi J. (2013). Can’t Get It Out of My Mind: Employee Rumination after Customer Mistreatment and Negative Mood in the Next Morning. Soc. Sci. Electron. Publ..

[31] Newman D.B., Nezlek J.B. (2017). Private self-consciousness in daily life: Relationships between rumination and reflection and well-being, and meaning in daily life. Personal. Individ. Differ..

[32] Nahum-Shani I., Henderson M.M., Lim S., Vinokur A.D. (2014). Supervisor support: Does supervisor support buffer or exacerbate the adverse effects of supervisor undermining?. J. Appl. Psychol..

[33] Boh W.F., Wong S.S. (2015). Managers versus co-workers as referents: Comparing social influence effects on within- and outside-subsidiary knowledge sharing. Organ. Behav. Hum. Decis. Processes.

[34] Peker M., Doğru O.C., Meşe G. (2022). Role of Supervisor Behavioral Integrity for Safety in the Relationship between Top-Management Safety Climate, Safety Motivation, and Safety Performance. Saf. Health Work..

[35] Park I.-J., Zhu D., Doan T., Kim P.B. (2021). Stay away from fickle supervisor! Supervisors’ behavioral fluctuation diminishing the effect of job embeddedness on employees’ service behavior. Int. J. Hosp. Manag..

[36] Maas V.S., Yin H. (2022). Finding partners in crime? How transparency about managers’ behavior affects employee collusion. Account. Organ. Soc..

[37] Watkins E.R. (2015). Psychological treatment of depressive rumination. Curr. Opin. Psychol..

[38] Fuente-Anuncibay R., González-Barbadillo N., Ortega-Sánchez D., Ordóez-Camblor N., Pizarro-Ruiz J.P. (2021). Anger Rumination and Mindfulness: Mediating Effects on Forgiveness. Int. J. Environ. Res. Public Health.

[39] Zuzama N., Fiol-Veny A., Roman-Juan J., Balle M. (2020). Emotion Regulation Style and Daily Rumination: Potential Mediators between Affect and Both Depression and Anxiety during Adolescence. Int. J. Environ. Res. Public Health.

[40] Gong T., Ren Y., Wu J., Jiang Y., Hu W., You J. (2019). The associations among self-criticism, hopelessness, rumination, and NSSI in adolescents: A moderated mediation model. J. Adolesc..

[41] Bushman B.J. (2016). Does Venting Anger Feed or Extinguish the Flame? Catharsis, Rumination, Distraction, Anger, and Aggressive Responding. Personal. Soc. Psychol. Bull..

[42] Ciesla J.A., Roberts J.E. (2007). Rumination, negative cognition, and their interactive effects on depressed mood. Emotion.

[43] Frone M.R. (2015). Relations of negative and positive work experiences to employee alcohol use: Testing the intervening role of negative and positive work rumination. J. Occup. Health Psychol..

[44] Kim Y.J., Kang S.W. (2021). An Analysis of the Relationship between the Modified Theory of Planned Behavior and Leisure Rumination of Korean Employees. Int. J. Environ. Res. Public Health.

[45] Smith M.B., Webster B.D. (2017). A moderated mediation model of Machiavellianism, social undermining, political skill, and supervisor-rated job performance. Personal. Individ. Differ..

[46] Quade M.J., Greenbaum R.L., Mawritz M.B. (2018). “If Only My Coworker Was More Ethical”: When Ethical and Performance Comparisons Lead to Negative Emotions, Social Undermining, and Ostracism. J. Bus. Ethics.

[47] Duffy M.K., Ganster D.C., Pagon M. (2002). Social Undermining in the Workplace. Acad. Manag. J..

[48] Duffy M.K., Ganster D.C., Shaw J.D., Johnson J.L., Pagon M. (2006). The social context of undermining behavior at work. Organ. Behav. Hum. Decis. Processes.

[49] Duffy M.K., Scott K.L., Shaw J.D., Tepper B.J., Aquino K. (2012). A Social Context Model of Envy and Social Undermining. Acad. Manag. J..

[50] Jung H.S., Yoon H.H. (2019). The effects of social undermining on employee voice and silence and on organizational deviant behaviors in the hotel industry. J. Serv. Theory Pract..

[51] Greenbaum R.L., Mawritz M.B., Piccolo R.F. (2012). When Leaders Fail to “Walk the Talk” Supervisor Undermining and Perceptions of Leader Hypocrisy. J. Cost Manag..

[52] Reh S., Tröster C., Quaquebeke N.V. (2018). Keeping (Future) Rivals Down: Temporal Social Comparison Predicts Coworker Social Undermining via Future Status Threat and Envy. J. Appl. Psychol..

[53] Xie Y., Kong Y., Yang J., Chen F. (2019). Perfectionism, worry, rumination, and distress: A meta-analysis of the evidence for the perfectionism cognition theory. Personal. Individ. Differ..

[54] Yang F.X., Xu Y.H., Wong I.A. (2021). Too close to work together? Identity conflicts induced by coworker friendships in cyberspace. Int. J. Hosp. Manag..

[55] Ayoko O.B., Callan V.J. (2010). Teams’ reactions to conflict and teams’ task and social outcomes: The moderating role of transformational and emotional leadership. Eur. Manag. J..

[56] Lakey B., Orehek E. (2011). Relational regulation theory: A new approach to explain the link between perceived social support and mental health. Psychol. Rev..

[57] Zetsche U., Bürkner P., Schulze L. (2018). Shedding light on the association between repetitive negative thinking and deficits in cognitive control–a meta-analysis. Clin. Psychol. Rev..

[58] Wang Q., Tu R., Jiang Y., Hu W., Luo X. (2022). Teasing and Internet Harassment among Adolescents: The Mediating Role of Envy and the Moderating Role of the Zhong-Yong Thinking Style. Int. J. Environ. Res. Public Health.

[59] Ganegoda D.B., Bordia P.I. (2018). Can Be Happy for You, but Not All the Time: A Contingency Model of Envy and Positive Empathy in the Workplace. J. Appl. Psychol..

[60] Feng W., Irina Y.Y., Yang M.X., Yi M. (2021). How being envied shapes tourists’ relationships with luxury brands: A dual-mediation model. Tour. Manag..

[61] Tai K., Narayanan J., McAllister D.J. (2012). Envy as Pain: Rethinking the Nature of Envy and Its Implications for Employees and Organizations. Acad. Manag. Rev..

[62] Kim E., Glomb T.M. (2014). Victimization of high performers: The roles of envy and work group identification. J. Appl. Psychol..

[63] Lee K.Y., Duffy M.K., Scott K.L., Schippers M.C. (2017). The experience of being envied at work: How being envied shapes employee feelings and motivation. Pers. Psychol..

[64] Kim S., O’Neill J., Cho H.M. (2010). When does an employee not help coworkers? The effect of leader–member exchange on employee envy and organizational citizenship behavior. Int. J. Hosp. Manag..

[65] Ye Y., Lyu Y., Kwan H.K., Chen X., Cheng X.-M. (2021). The antecedents and consequences of being envied by coworkers: An investigation from the victim perspective. Int. J. Hosp. Manag..

[66] Habimana E., Massé L. (2000). Envy manifestations and personality disorders. Eur. Psychiatry.

[67] Parrott W.G., Rodriguez Mosquera P.M. (2008). On the Pleasures and Displeasures of Being Envied. Envy.

[68] Silver M., Sabini J. (1978). The Perception of Envy. Soc. Psychol..

[69] Woerkom M.V., Bakker A.B., Nishii L.H. (2016). Accumulative job demands and support for strength use: Fine-tuning the JD-R model using COR theory. Acad. Manag. Annu. Meet. Proc..

[70] Chan M.E., Mcallister D.J. (2014). Abusive supervision through the lens of employee state paranoia. Acad. Manag. Rev..

[71] Liu F., Liu D., Zhang J., Ma J. (2019). The relationship between being envied and workplace ostracism: The moderating role of neuroticism and the need to belong. Personal. Individ. Differ..

[72] Ahlich E., Herr J.B., Thomas K., Segarra D.T., Rancourt D. (2020). A test of the stress-buffering hypothesis of social support among bariatric surgery patients. Surg. Obes. Relat. Dis..

[73] Beehr T.A., Farmer S.J., Glazer S., Gudanowski D.M., Nair V.N. (2003). The enigma of social support and occupational stress: Source congruence and gender role effects. J. Occup. Health Psychol..

[74] Jiang W., Wang L., Lin H. (2016). The role of cognitive processes and individual differences in the relationship between abusive supervision and employee career satisfaction. Personal. Individ. Differ..

[75] Arnau-Sabatés L., Gilligan R. (2020). Support in the workplace: How relationships with bosses and co-workers may benefit care leavers and young people in care. Child. Youth Serv. Rev..

[76] Grobelna A. (2020). Emotional exhaustion and its consequences for hotel service quality: The critical role of workload and supervisor support. J. Hosp. Mark. Manag..

[77] Eisenberger R., Cummings J., Armeli S., Lynch P. (1997). Perceived organizational support, discretionary treatment, and job satisfaction. J. Appl. Psychol..

[78] Eisenberger R., Stinglhamber F., Vandenberghe C., Sucharski I.L., Rhoades L. (2002). Perceived supervisor support: Contributions to perceived organizational support and employee retention. J. Appl. Psychol..

[79] Rhoades L., Eisenberger R., Armeli S. (2001). Affective commitment to the organization: The contribution of perceived organizational support. J. Appl. Psychol..

[80] Cheng-Ling T. (2012). The Relationships among Leader Social Support, Team Social Support, Team Stressors and Team Performance. Procedia-Soc. Behav. Sci..

[81] Rachel A., Thomas E., Tim R. (2018). Organizational stressors, social support, and implications for subjective performance in high-level sport. Psychol. Sport Exerc..

[82] Mao Z., Duan Y., Yao Y., Huo J. (2021). The moderating effect of average wage and number of stores on private label market share: A hierarchical linear model analysis. J. Retail. Consum. Serv..

[83] Raudenbush S.W., Bryk A.S. (2002). Hierarchical Linear Models: Applications and Data Analysis Methods.

[84] Kreft I.G. (1996). Are Multilevel Techniques Necessary? An Overview, Including Simulation Studies.

[85] Zhang X., Wang J.Y. (2010). Research on sample size problem of layered linear model. Stat. Decis..

[86] Vecchio R. (2005). Explorations in employee envy: Feeling envious and feeling envied. Cogn. Emot..

[87] Zimet G.D., Dahlem N.W., Zimet S.G., Farley G.K. (1988). The multidimensional scale of perceived social support. J. Personal. Assess..

[88] James L.R. (1982). Aggregation bias in estimates of perceptual agreement. J. Appl. Psychol..

[89] Kelarestaghi K.B., Ermagun A., Heaslip K. Cycling Usage and Frequency Determinants in College Campuses. Proceedings of the Transportation Research Board 97th Annual Meeting.

[90] Yalvaç E.B.K., Gaynor K. (2021). Emotional dysregulation in adults: The influence of rumination and negative secondary appraisals of emotion. J. Affect. Disord..

[91] Okihara T., Koizumi K., Takahashi H., Suzuki M., Makita S. (2022). Correlation between Psychological Rumination and Symptoms of Traumatic Stress in Patients with Mild Paralysis in Acute Phase of Stroke: A Cross-sectional Study. J. Affect. Disord. Rep..

[92] Spinhoven P., van Hemert A.M., Penninx B.W. (2019). Repetitive negative thinking as a mediator in prospective cross-disorder associations between anxiety and depression disorders and their symptoms. J. Behav. Ther. Exp. Psychiatry.

[93] Ghaith S.M., Al-Baddareen G.S., Ali T., Akour M.M. (2020). Perceived social support among widowed women in Jordan: An exploratory study. Women’s Stud. Int. Forum.

[94] Wenninger H., Cheung C.M., Chmielinski M. (2021). Understanding envy and users’ responses to envy in the context of social networking sites: A literature review. Int. J. Inf. Manag..

[95] Yang F., Wen D. (2021). Combating workplace loneliness climate and enhancing team performance: The roles of leader humor and team bureaucratic practices. J. Bus. Res..

[96] Chervoni-Knapp T. (2021). Mitigating Strategies for Interpersonal Workplace Conflict and Violence. J. Radiol. Nurs..

